# Unreported Rift Valley fever virus circulation during 2023–2024 El Niño event detected by slaughterhouse-based surveillance in southern Kenya

**DOI:** 10.1038/s41598-026-44706-y

**Published:** 2026-03-19

**Authors:** Keli Nicole Gerken, Abraham Rereu, Victor Mutai, Alice Kiyong’a, Richard Rasto Olubowa, Elizabeth Anne Jessie Cook, Matthew Baylis, Reuben Mwangi, Frederick Sururu, Andrew Stringer, Eric M. Fèvre

**Affiliations:** 1 Institute of Infection, Veterinary, and Ecological Sciences, University of Liverpool, Liverpool, UK; 2 International Livestock Research Institute, Nairobi, Kenya; 3 Kajiado County Department of Veterinary Services, Oloitokitok Sub-County, Kajiado, Kenya

**Keywords:** Diseases, Medical research, Microbiology

## Abstract

Rift Valley fever (RVF) is a zoonotic arbovirus, and livestock cases are often underreported in endemic countries due to reliance on passive clinical surveillance. During the 2023–2024 El Niño event, Kenya experienced widespread flooding, but no RVF outbreaks were reported in the southern regions. We implemented slaughterhouse-based surveillance in southern Kenya from May 2023—June 2024, using five consecutive cross-sectional surveys. Cattle, sheep, and goats were tested for anti-RVFV IgG and IgM antibodies, with concurrent recording of post-mortem lesions. Using age estimates from dentition, catalytic models estimated the force of infection (FOI) over time and spatial analysis assessed the Loitokitok sub-county for hotspots. Among 955 animals, 10.2% were IgG-positive, with seroprevalence and FOI increasing after El Niño rains, reaching 22.6% by May 2024. Six animals (0.6%) were IgM-positive, indicating recent infection, with cases detected in 3/5 sampling periods, including before the rains. All recently infected IgM-positive animals were deemed fit for slaughter and none had lesions. Adult animals in endemic areas, void of clinical and pathological signs, may therefore play a role in the silent spread and maintenance of RVFV. Slaughterhouse-based surveillance offers a practical and scalable platform for improving RVF detection and monitoring livestock in endemic regions.

**Supplementary Information:**

The online version contains supplementary material available at 10.1038/s41598-026-44706-y.

## Introduction

Rift Valley fever virus (RVFV) is a climate-sensitive zoonosis with serious implications for livestock health, trade, and public health^1–3^. The virus can be transmitted by numerous different mosquito vectors and infect multiple mammalian hosts, providing it with a high potential for transboundary spread^4–6^. RVFV is classically understood as an outbreak-focused disease triggered by heavy rainfall, but there is substantial evidence to suggest that transmission also occurs during so-called “interepidemic” periods^7–9^. A comprehensive analysis across East Africa indicates that indeed, most recent RVF confirmed cases have been associated with smaller localized outbreak clusters, rather than large outbreaks^10^. The risk of transboundary spread via livestock during these quieter periods of transmission is poorly understood, and adult animals may show no clinical signs, highlighting the need for more sensitive and systematic approaches to surveillance^11,12^.

Despite increasing recognition of endemic disease patterns in East Africa, RVF surveillance systems remain largely reactive, focused on outbreak prediction, and reliant on passive clinical reporting. Even in instances where early warning alerts prompt more active case finding at the community-level, there can be no PCR confirmed cases, highlighting the narrow window of detection^13^. Sparse interepidemic data and negative passive surveillance efforts have contributed to an incomplete understanding of the true risk profile of endemic RVF and blurred the boundaries of what constitutes an outbreak^14–16^. This discourages reporting and undermines consistent implementation of trade restrictions between countries with differing surveillance strategies^17^. Understanding the extent and seasonal pattern of endemic RVFV transmission is directly relevant to assessing and mitigating the risk of spread within and between countries.

As with other endemic diseases, recognising endemic RVF is complicated by a plethora of factors present in human and animal health systems including clinical syndromes overlapping with other diseases, awareness, and that most clinical manifestations are mild and self-limiting^18,19^. In Kenya, a large proportion of livestock are raised extensively in semi-pastoral and pastoral settings. These outbreak-prone areas often have high livestock density but low human population and infrastructure density, which makes routine sampling of live animals at a landscape scale impractical beyond research studies or targeted outbreak investigations^20,21^.

Where sampling occurs is just as important as when it occurs, as transmission intensity can vary significantly across relatively small geographical areas. A study in Tanzania found that the force of infection (FOI), defined as the annual probability that a susceptible animal becomes infected with RVFV, varied over 20-fold across study villages despite no major ecological or cultural differences, suggesting highly localized transmission hotspots^22^. Like outbreak data, this heterogeneity complicates interpretation of patchy cross-sectional studies and reinforces the need for a more systematic, active, and year-round surveillance approach^16^. Identifying where transmission is occurring, what factors are driving it, and when risk increases is critical to improving RVF management in endemic areas and limiting the wider spread of disease.

Controlling disease in livestock can reduce the public health impact and limit further spread of RVFV; however, detecting is a prerequisite for control^5^. If abortion storms are not recognisable because of smaller case clusters and a higher level of baseline herd immunity, slaughterhouse-based testing may provide one of the few remaining entry points for early detection. In Kenya, meat inspectors are required to be present at all slaughterhouses and conduct routine active surveillance for specific diseases and assess meat to be fit for consumption^23^. Slaughterhouses have, more generally, been identified as key sites for surveillance of this kind of One Health problem^24^.

Identification of pathognomonic lesions during routine post-mortem meat inspections at slaughterhouses can provide a valuable screening tool to monitor disease risk. A prevalent example is liver fluke infection (*Fasciola gigantica*) which causes distinct lesions, including enlarged bile ducts, fibrotic tracks, and “pipestem” fibrosis^25^. Liver fluke infections are visually detectable and widespread across Kenya^26^, and systematic recording of lesions at slaughter has led to assessments of economic losses^27^. In contrast, zoonotic diseases such as brucellosis rarely produce visible post-mortem lesions in livestock hosts, making diagnosis heavily reliant on quality laboratory tests^28^. Post-mortem lesions associated with RVFV have been described in lambs, the most severely impacted species, infected during outbreaks^29^. In these young and vulnerable hosts, multi-focal necrotizing hepatitis was common, followed by evidence of renal injury, neither of which are specific to RVFV infection. While sub-clinical and silent RVFV infections has been suggested in adult ruminants, especially indigenous breeds^30^, it remains unclear whether these animals develop subtle and consistent lesions that could serve as reliable markers for slaughterhouse-based surveillance.

The 2023–2024 El Niño event in East Africa led to widespread flooding in Kenya and early warning systems predicted RVF outbreaks across the country and region^31^. Yet, no RVF outbreaks were reported in southern Kenya, despite favourable conditions, and the cases that occurred in the northernmost County, Marsabit, were understood as the extent of RVF transmission in Kenya^32^. The present study builds on previous efforts to integrate RVFV testing of livestock at slaughter and provides a framework in which this can be implemented over years to monitor endemic transmission risk^33,34^. We link post-mortem lesion identification to RVFV testing for evidence of prior exposure and recent livestock infection. We then use age-structured data to calculate the FOI over tim, and conduct spatial analyses to understand geographical risk for lesions and RVFV exposure in livestock.

## Results

Overall, 955 animals were sampled over five sampling periods (A-E) (Table 1). The anti-RVFV IgG seroprevalence was 10.2% (97/955). Seroprevalence varied significantly by sampling period (*p* < *0.0001*), with the lowest (2.3%, 4/178) recorded in sampling period C (November–December 2023) at the end of a severe drought period and start of the El Niño rains. The highest seroprevalence was then recorded at the end of the study (period E) in May 2024 (22.6%, 42/186) (Table 1). The most remote slaughterhouse we sampled at, Rombo, had the lowest overall seroprevalence (3.2%), and did not supply any recently infected (IgM-positive) animals to this study. Animals purchased at markets had higher seropositivity than those that were presented directly from their households, but this difference was not significant (*p* =*0.08*).

**Table 1:** Descriptive statistics of prior exposure to and recent infection with RVFV.

**Variable**		**Total samples**	**IgG + (%)**	**P-value**	**IgM + (%)**	**P-value**
	Total samples	955	97 (10.2)		6 (0.6)	
Species	Cattle	405	41 (10.1)	0.85	1 (0.2)	0.43
	Sheep	275	26 (9.5)		2 (0.7)	
	Goats	275	30 (10.9)		3 (1.1)	
SH	Rombo	103	4 (3.9)	0.09	0	1.0
	Loitokitok	205	25 (12.2)		1 (0.5)	
	Kimana	623	64 (10.3)		5 (0.8)	
	Ilasit	24	4 (16.7)		0	
Sampling period	A: May 2^nd^- May 26^th^ 2023	206	22 (10.7)	< 0.0001	1 (0.5)	0.11
	B: July 31^st^ – Aug 16^th^ 2023	213	18 (8.5)		0	
	C: Nov 20^th^ – Dec 14^th^ 2023	178	4 (2.3)		3 (1.7)	
	D: Jan 17^th^ – Feb 15^th^ 2024	172	11 (6.4)		2 (1.2)	
	E: May 14^th^ – June 21^st^ 2024	186	42 (22.6)		0	
Estimated age	Less than 1.5 years	26	2 (7.7)	0.003	0	0.82
	1.5–2 years	58	4 (6.9)		0	
	2–3 years	194	12 (6.2)		2 (1.0)	
	3–4 years	357	32 (9.0)		3 (0.8)	
	> 4 years old	320	47 (14.7)		1 (0.3)	
Number in group at slaughter	1	819	77 (9.4)	0.68	4 (0.5)	0.07
	2	98	18 (18.4)		1 (1.0)	
	3	15	1 (6.7)		0	
	4	9	0		0	
	5	5	1 (20.0)		0	
	6	7	0		1 (14.3)	
	NA	2	0		0	
Arrived in vehicle	Yes	33	5 (15.2)	0.50	0	1.0
	No	918	92 (10.0)		6 (0.7)	
	NA	4	1			
Purchased at market	Yes	740	84 (11.4)	0.08	5 (0.7)	1.0
	No	214	13 (6.1)		1 (0.5)	
	NA	1	0		0	

SH: Slaughterhouse, P = Chi-square test for categorical variables, Cochran-Armitage for ordinal variables (age category and number received with), Fishers Exact test for small samples such as IgM results. The origin locations above have combined the reported location or sub-location in the sub-county.

In adjusted multivariable analysis, sampling period C (OR = 0.19, *p* = *0.004*) was associated with lower odds of exposure and period E had higher odds (OR = 2.73, *p* = *0.002*). Higher odds of IgG seropositivity were also associated with the Loitokitok slaughterhouse (OR = 3.74, *p* = *0.03*) and passing through a market (OR = 2.20, *p* = *0.04*) before slaughter. In contrast, the animal’s species, age, and group size were not independently associated with IgG seropositivity after adjustment.

### Age of slaughtered animals

**Fig. 1:**
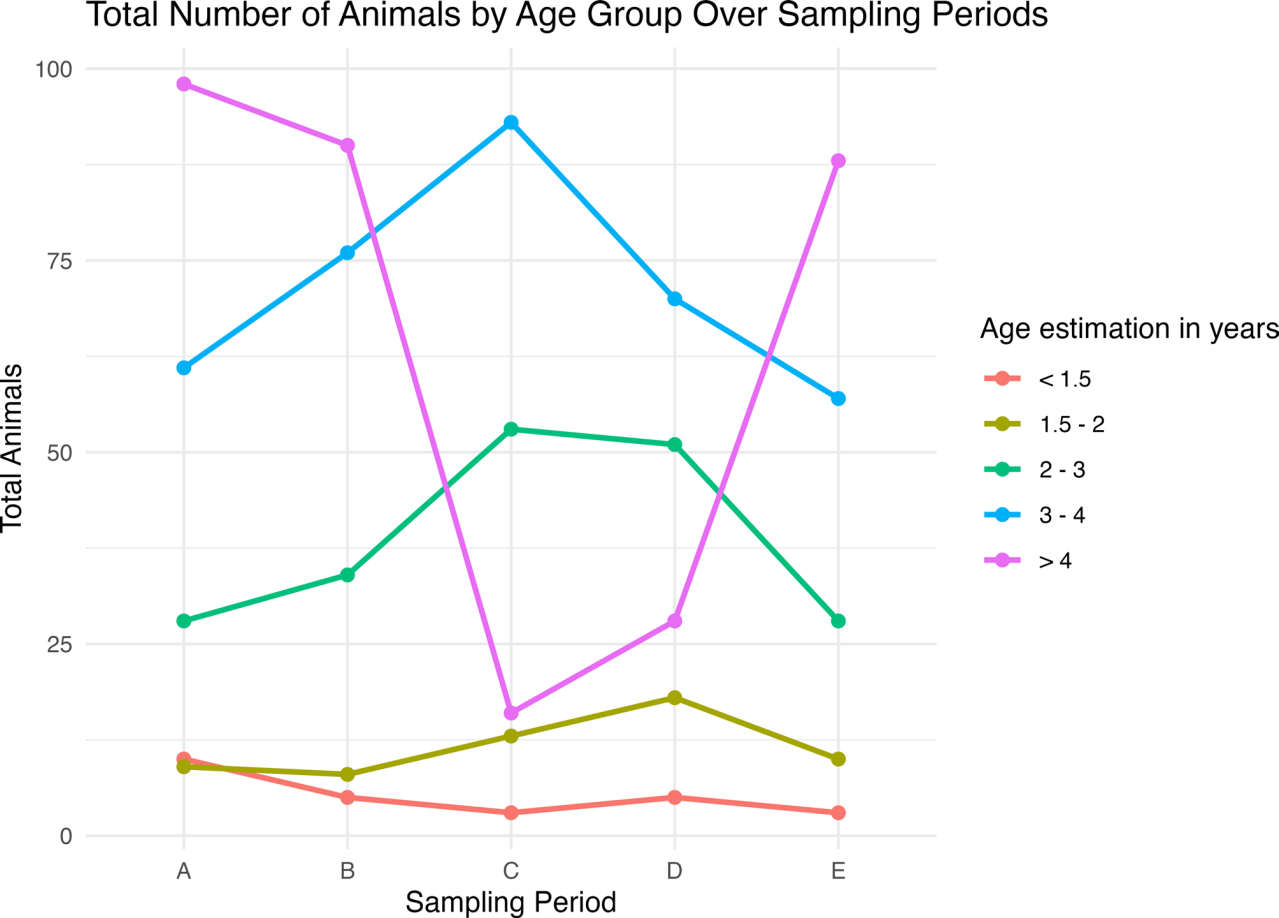
Total number of animals sampled per age group over sampling periods. Age distribution of slaughtered animals across five sampling periods (A–E). Age was estimated by dentition and frequencies are pooled across all slaughterhouse sampling sites.

Although age was associated with RVFV IgG seropositivity in bivariable analysis (Table 1, *p* = *0.003*), this effect was not retained when accounting for when (sampling period) and where (slaughterhouse) animals were sampled. Age distributions varied substantially over the study period (Fig. 1), with period C having the lowest proportion of older animals. Further visualization of age-stratified seroprevalence plots confirmed that all age groups had decreased seroprevalence in period C.

### Summary of recent and acute infections (IgM-positive cases)

This study identified six IgM-positive animals (0.6%; 6/955), indicating recent RVFV infection, over the full study period. These animals were all greater than two years old and there was not a statistically significant association between IgM positivity and age (*p* = *0.81*). All species (cattle n = 1, goats n = 3, sheep n = 2) were represented in the IgM-positive animals, and three of the five sampling periods (A, C, D) had at least one positive animal. The highest number of IgM-positive animals (n = 3) were identified after the start of the 2023 El Niño rains (period C), followed by period D after three months of rain (n = 2). None of the IgM-positive animals had any post-mortem lesions. The six IgM-positive samples were tested by rt-PCR, and all were confirmed to be negative.

Only one of the six IgM-positive animals was slaughtered at the Loitokitok slaughterhouse in sampling period A, originating from a village less than 7 km from Kimana town center. The other five IgM-positive animals were all reported to be from the greater Kimana area and slaughtered at the Kimana slaughterhouse in period C (n = 3) and D (n = 2). Kimana slaughterhouse also supplied the most animals to this study, so there was not a statistically significant difference in IgM detection between slaughterhouses (*p* = *1.00*). There were no common predictor variables for IgG and IgM positivity.

### Force of infection over time

**Fig. 2:**
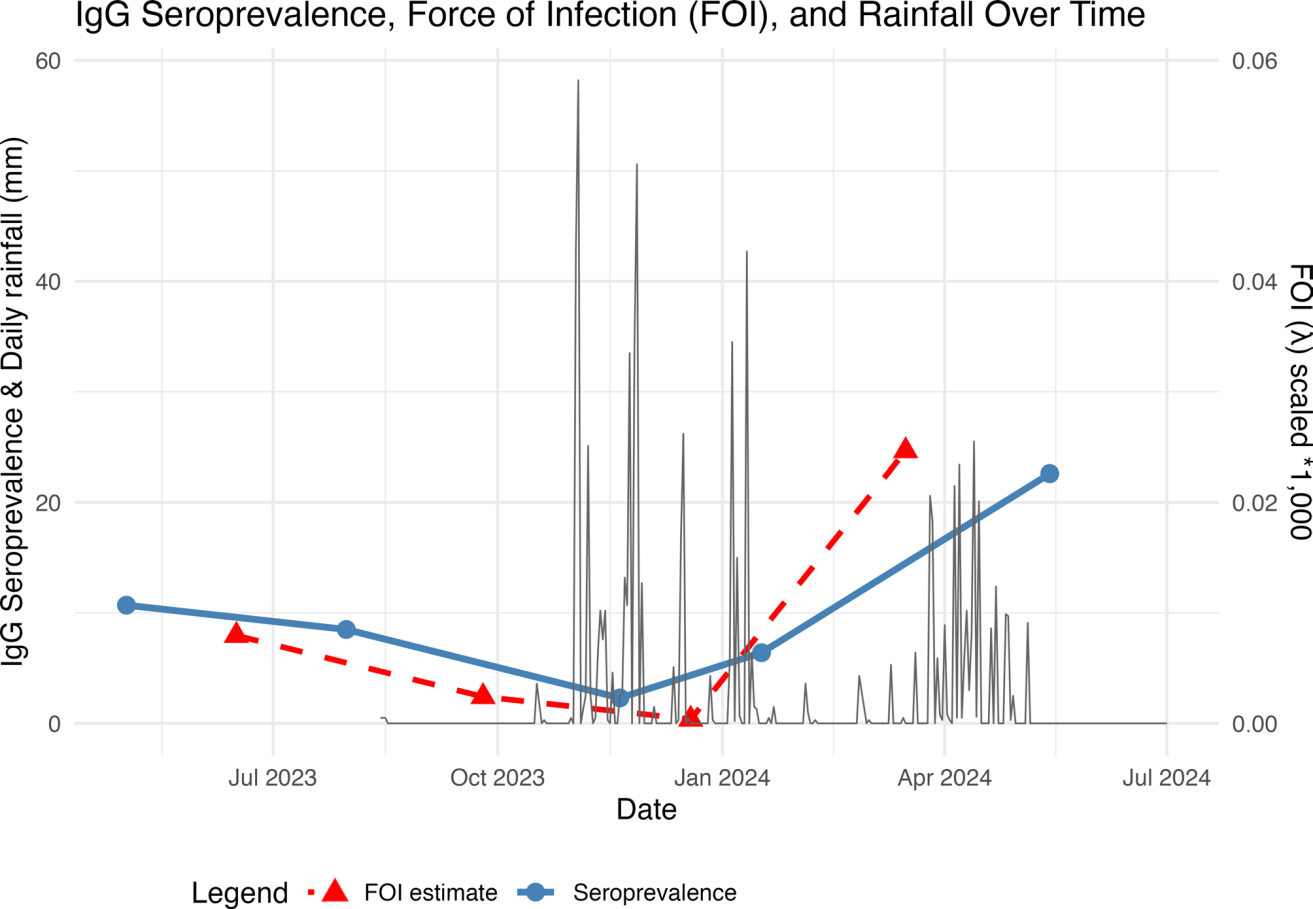
Temporal trends in IgG seroprevalence, estimated force of infection (FOI), and daily rainfall. Given that the FOI estimates were for combined sampling periods, the marker has been placed equidistant between the two sampling points. The rainfall data (grey line) has been obtained from our Ecowitt HP2551 weather station at the Kimana Health Centre in Kimana town and demonstrates the onset of the 2023 El Nino rainfall in November. Weather data were not available from this station prior to August 2023.

The force of infection for entire sampling period was 0.016 per year which means on average, 1.6% of animals in the study area become infected each year. Due to limited sample sizes in the youngest age group and skewed age distribution in periods 3 and 4, FOI could not be reliably estimated for each sampling period independently. FOI calculations for the for four combined time periods overlaid with seroprevalence and rainfall are displayed in Table 2 and Fig. 2.

**Table 2:** Summary of FOI estimates for each sampling period combination and overall.

Sampling periods	Estimated Annual Proportion Infected (%)
Period 1 and 2	0.80
Period 2 and 3	0.24
Period 3 and 4	0.04
Period 4 and 5	2.46
Period 1–5 (All data)	1.60

FOI estimates are expressed as average annual rates, consistent with the age variables units (years), even though these were age estimates based on dentition. To achieve estimated annual proportions infected, the calculated FOI value is multiplied by 100 (e.g. 0.08% corresponds to a calculated FOI of 0.0080).

### Spatial distribution of RVFV exposure

**Fig. 3:**
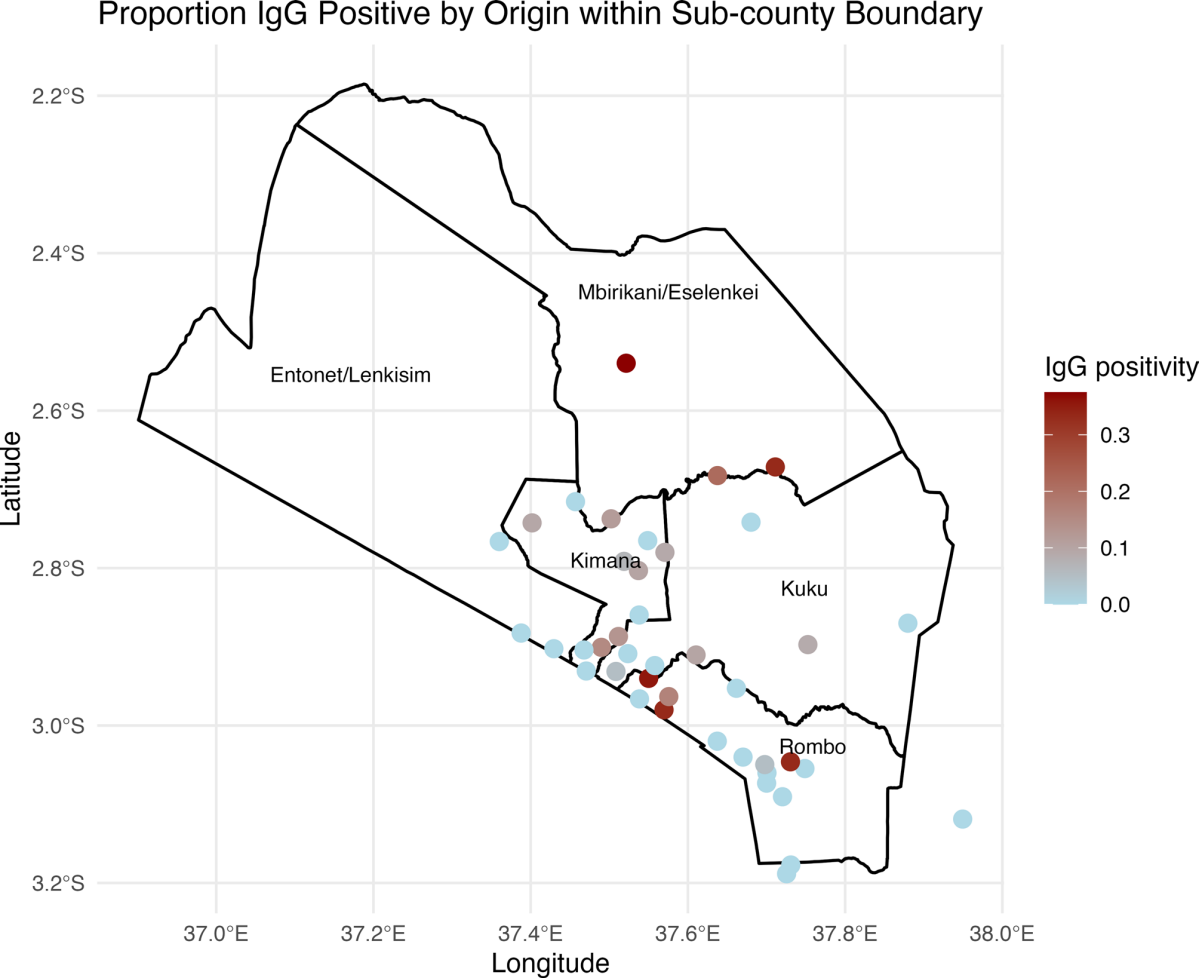
Proportion of IgG positivity aggregated by the reported origin location across wards within the Loitokitok sub-county boundary. Proportion of RVFV IgG seropositive animals by reported origin within Loitokitok sub-county. Each point represents a sampled origin pooled across all surveys, with color intensity indicating the proportion IgG-positive (scale from 0 to 0.3). Ward boundaries are outlined in black using open-source GADM level 3 data for Kenya.

While most of the IgM-positive recent infections (5/6) were reported to be from the Kimana area, the proportion of IgG seropositivity was evenly distributed throughout the origin locations (Fig. 3).

Origin location data was available for 854 samples (89.4% of total samples, 854/955) and represented 42 different locations. We filtered locations to those with at least give samples in at least one sampling period, which resulted in 17 locations and these locations were used to carry out logistic regression to determine if any specific locations had a significant impact on the period prevalence. Across all five sampling periods, the intercept of models was statistically significant (*p* = *0.002*), but none of the individual location coefficients differed significantly from the reference category. Similarly, in each period-specific model, location was not a significant predictor of RVFV IgG seropositivity. Likelihood ratio tests comparing models with and without the location term further confirmed no evidence of spatial variation in IgG positivity across the sampled locations.

Additionally, there was no evidence of global spatial clustering of IgG-positive animals according to the join count test, nor evidence of positive spatial autocorrelation (*p* = *0.50*). Join count tests were not conducted for each sampling period as origin locations were more sparse and rarely within 5,000 m.

### Lesions identified and associations with RVFV exposure

Overall, this study identified at least one lesion in at least one organ in 15.0% (143/955) of animals, commonly affecting the liver (60.1%, 86/143), lung (46.2%, 66/143), and kidney (21.0%, 30/143), with some animals having multiple lesions. Cattle had the most lesions (70.0%, 110/143), followed by goats (13.3%, 19/143), and sheep (9.8%, 14/143), and most lesions were in animals greater than three years (75.5%, 108/143). Age overall was not significantly associated with having any lesion (*p* = *0.14*), except for a liver lesion (*p* = *0.03*). A summary of the specific lesions for each organ system is presented in Supplementary file S1.

We also examined the statistical relationships between RVFV exposure (IgG-positive) and these major lesions. The only lesion that was significantly associated with prior exposure to RVFV was cysts in the lung (*p* = *0.01*). Visually, positive cases clustered north of Kimana town, but lung cysts were not spatially clustered as a single variable (*p* = *0.93*). In fact, none of the major lesions we identified across the study site had significant spatial clustering according to the join count test.

### Origin locations and discrepancy in lesion reporting across slaughterhouses

**Fig. 4:**
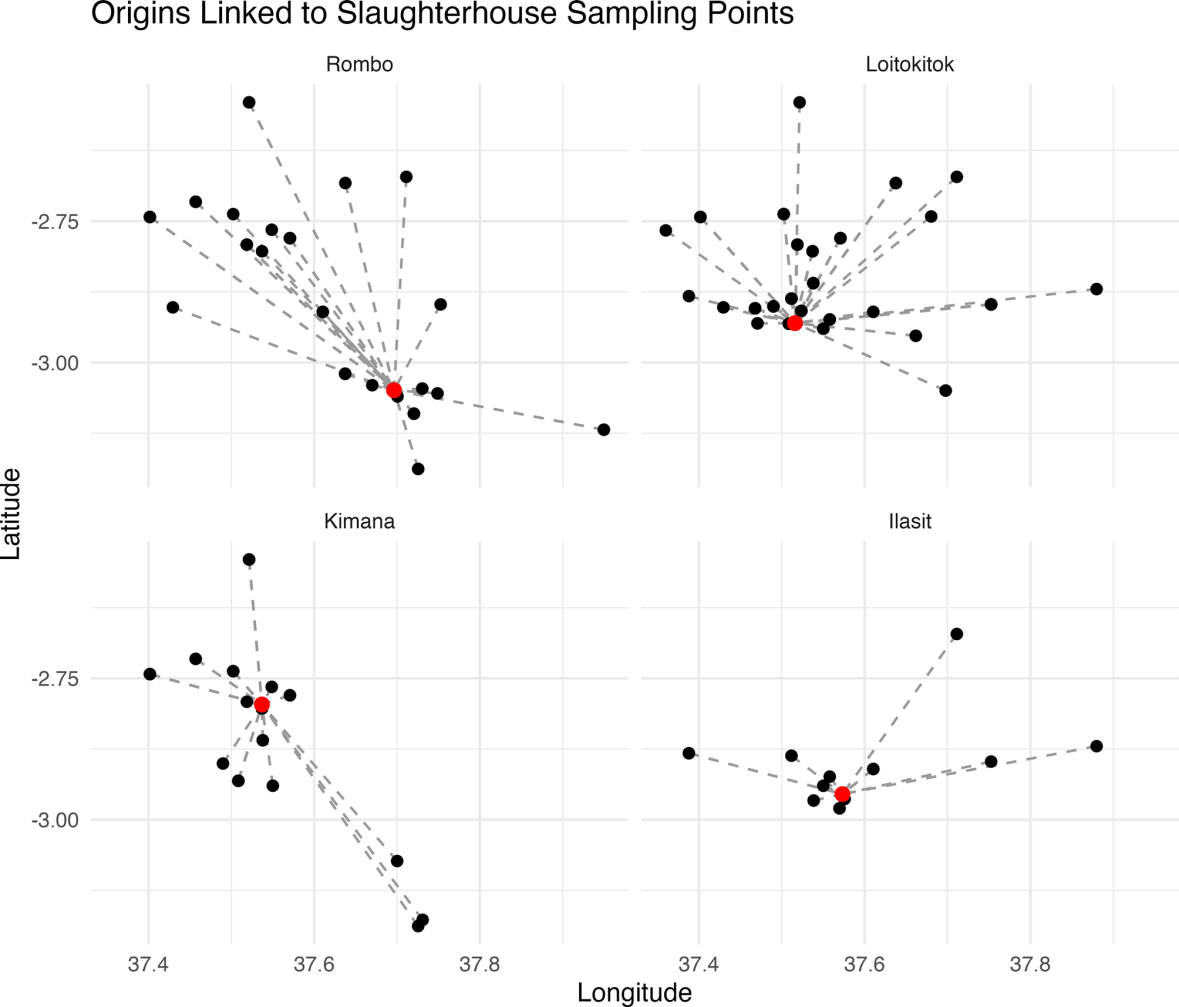
Slaughterhouse locations and the origins that supplied them. Reported animal origins (black dots) and slaughterhouse locations (red dots) in Loitokitok sub-county. Each panel shows one slaughterhouse, with dashed lines linking sampled animals’ reported origins to the corresponding slaughterhouse where they were processed.

All slaughterhouses covered a broad range of origin locations across the study site (Fig. 4), and animal origins differed significantly between slaughterhouses (X^2^ = 1698.4, *p* < *0.001*), likely driven by the large number of Kimana-sourced animals slaughtered at the Kimana slaughterhouse.

All lesion types were evenly distributed across the reported origin locations with no spatial auto correlation; however, there was a significant discrepancy in the proportion of animals with lesions identified at each slaughterhouse (*p* < *0.001*). The Rombo and Loitokitok slaughterhouse, where the same veterinarian works, had a similar rate of detection, but at the Kimana slaughterhouse—where the highest volume of animals per day are slaughtered— only 6.1% (38/623) of animals had a lesion. Interestingly, most kidney lesions in this study were identified at the Loitokitok slaughterhouse with 26.8% (55/205) of sampled animals having a kidney lesion.

## Discussion

We demonstrate evidence of low-level RVFV transmission in livestock throughout the year in a high-risk area of Kenya, in the absence of reported livestock outbreaks, even following heavy rainfall and flooding. This finding is consistent with the hypothesis of ongoing, endemic transmission of RVFV in this ecosystem. Transmission intensity increased after heavy 2023–2024 El Niño rainfall (Fig. 2), during which large parts of Kenya experienced flooding, but this rise remained below the passive detection threshold, and no abortion storms were reported. Our study findings point towards three major implications for endemic RVF transmission: 1) Active testing in livestock is needed to detect and monitor silent, year-round transmission; 2) Recently infected adult animals may not have clinical signs or gross post-mortem lesions; and 3) Slaughterhouse-based surveillance offers a scalable platform for detecting RVFV circulation, but diagnostics are essential. Each of these factors is relevant to the transboundary spread potential of RVF, as animals transported for slaughter may contribute to the unrecognized movement of the virus across regions outside of outbreak periods when awareness is low.

Our IgM detections add to the growing evidence that transmission occurs year-round, not just after the rains. Another study in northern Kenya two years prior reported a comparable IgM detection rate of 0.4%, but a higher IgG seroprevalence of 21.7%, compared to our 0.6% and 10.2%, respectively^35^. Detection of IgM and viraemia overlap only briefly, 4–14 days post infection^2^, and none of the IgM-positive samples from our study were rt-PCR positive. Spatially, most (5/6) IgM-positive animals originated from the greater Kimana area, suggesting localized recent exposure, but IgG seroprevalence was not clustered, highlighting the need for longitudinal surveillance to detect both seasonal trends and geographic hotspots.

All six recently infected (IgM-positive) animals in our study were assessed antemortem and deemed fit for slaughter with no visible post-mortem lesions. This supports that adult animals may silently transmit and spread RVFV in endemic areas, aligning with models of silent carriers for other arboviruses^36,37^. Passive surveillance misses low-level transmission due to herd immunity building over time with repeated exposure and impacts the recognizability of livestock abortion events that are blurred with other aetiologies^38,39^. Adult animals, often involved in trade or migration in extensive production systems, are likely mediators of RVFV spread and maintenance but are also least likely to show clinical signs of RVF^40^. Given that arthropods are required for viral amplification in livestock^41^, understanding the temporal relationship between livestock movements and weather patterns that promote mosquito proliferation is critical to consider in assessments of transmission risk.

Slaughterhouses offer a scalable and underutilized platform for disease monitoring, particularly for generating widespread endemic case data to inform RVF risk analysis^24^. Integrating viral disease testing into these existing systems enables large-scale screening by leveraging livestock movement to slaughter to capture origin data and examine spatial risk^33^. In this study, blood sampling at slaughter detected both past exposure and recent infections. The full spectrum of RVF disease in adult animals in endemic countries remains vague and non-specific^42,43^, and we found no pathognomonic lesions associated with recent infections. This limits the utility of post-mortem observations for RVF detection and monitoring, and highlights the need to also capture species, age, and origin data alongside serological testing. Combined age and origin data allow for monitoring the force of infection (FOI), helping track transmission over time and space.

Routine meat inspection already provides active surveillance for important zoonotic parasitic diseases with pathognomonic lesions, but integration of diagnostic testing remains limited^44^. In our study, 15% of all animals had at least one lesion, many of which were non-specific. Without access to diagnostics, these tissues are condemned, preventing risk to consumers, but the aetiologies are unknown. For zoonoses with livestock reservoirs, visual inspection alone is often insufficient, and even when lesions are visible, sensitivity can be low^45^. We identified spatial clustering of RVF exposure and lung cysts consistent with *Echinococcus spp*., but the link is likely ecological as there are no known biological or epidemiological links between these pathogens. As diagnostic capacity continues to evolve in Kenya, integrating testing for RVFV and other pathogens into routine slaughterhouse workflows could overcome the logistical and design challenges of field-based studies and elevate meat inspectors as frontline One Health surveillance actors^46^.

This study has several limitations that can be included in efforts to scale the design. The post-mortem lesion identification varied between inspectors, possibly impacting consistency in reporting. Animals in the youngest age group were rarely sampled, and we had to adjust FOI estimates accordingly. In future studies, frequency differences across age groups should be monitored during data collection and considered in sample size calculations aimed at FOI estimation. Sampling intervals were also uneven, which may have influenced temporal comparisons. Although we used both IgG and IgM to assess exposure and recent infection, serum is suboptimal for rt-PCR confirmation^47^, and this was the only diagnostic sample we obtained; future studies should biobank both blood and serum. Further, antemortem inspections by a veterinarian is routine but was not systematically recorded in our dataset, so health assessment was subjective. Finally, previous work demonstrates that larger tertiary slaughterhouses in urban areas source animals from a broader origin catchment area^33^. To expand the utility of this slaughterhouse-based surveillance platform, sampling could be implemented at multiple scales and incorporate origin information from supplying marketplaces to specifically target animals from known high-risk areas.

Overall, our findings provide further evidence that slaughterhouse-based surveillance can fill critical gaps in current systems and should be considered a core component of endemic RVF monitoring frameworks.

## Conclusions: A call to action

In some areas, RVF exhibits an endemic transmission pattern, with active, undetected virus circulation in livestock. Here, the backwards pattern of waiting for human ‘index cases’ to trigger outbreak investigations helps perpetuate a hidden burden in livestock. Identifying and controlling RVF in animals remains critical for protecting human health, particularly as climate extremes intensify. The 2023–2024 El Niño event was among the most severe in Kenya’s recent history^48^, yet only one isolated outbreak cluster in the far north of the country was detected. There are clearly limitations in the effectiveness of current passive reporting surveillance. Our study demonstrates that active, slaughterhouse-based surveillance can detect ongoing transmission and increases in transmission intensity, even when clinical signs are absent.

Failing to recognize endemic RVFV circulation poses a hidden and unmeasured risk for transboundary spread through the movement of asymptomatic infected animals or animal products. Slaughterhouses offer a scalable platform for early detection of RVFV and other livestock pathogens across wide geographic areas using existing infrastructure and personnel^24^. They also allow integration of clinical, pathological, and serological data, and strengthening of diagnostic capacity at regional facilities such as the Loitokitok One Health lab that can improve hotspot detection and response.

Although abortion remains a hallmark of RVF in livestock, in the context of endemic RVF transmission, abortions may occur outside of large “abortion storms” and may go unrecognized amid the background of other causes. Thus, targeted testing of animals with recent abortions, especially rt-PCR on vaginal swabs, should complement slaughter-based surveillance. Overall, adapting surveillance to include detection of low-level endemic transmission is essential for developing more effective early warning systems and limiting transboundary spread potential. Such efforts would be a timely response to increase global health security in an era of increasingly unpredictable vector-borne and zoonotic disease threats like RVFV.

## Methods

This study aimed to characterize endemic transmission risk over time using consecutive cross-sectional surveys in slaughterhouses of Loitokitok sub-county, Kajiado County, Kenya to calculate exposure (IgG), recent infections (IgM), and transmission intensity (FOI). We also explore post-mortem lesions in the sample set and associations with RVFV occurence.

### Study site and sampling points

**Fig. 5:**
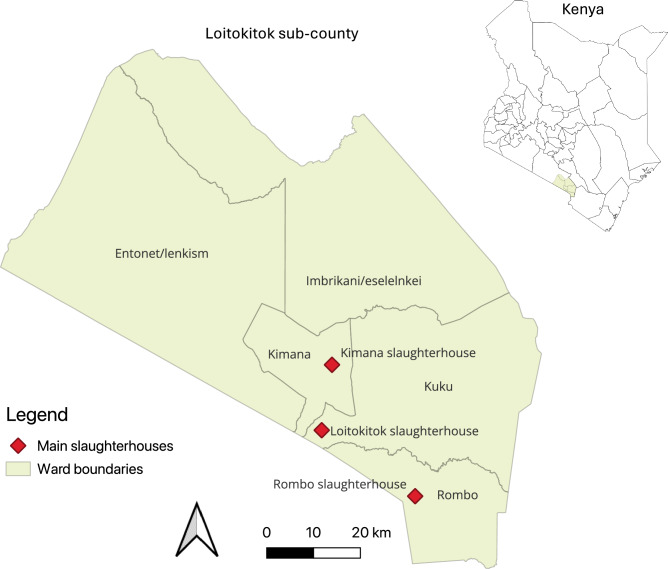
Map of the study site wards and slaughterhouse sampling point locations. Map of the study site in Loitokitok sub-county, Kajiado County, Kenya, showing the three main slaughterhouses (red diamonds) and ward boundaries (shaded areas). Inset shows the location of Loitokitok within Kenya. This map was created in QGIS software (https://qgis.org/) version 3.42 Münster using GADM open-source shape files for administrative boundaries of Kenya https://gadm.org/.

In the 2019 national census, Loitokitok sub-county (Kajiado County, Kenya) was home to 191,846 people across five total wards, sparsely populated in the north and Kimana, Loitokitok, and Rombo as larger towns in the south (Fig. 5). The region is a traditionally pastoral area that has undergone rapid growth and expansion of crop farming in the past decades^49^. In Kenya, most (80–90%) red meat consumption originates from pastoral systems, and while home slaughter does occur, the majority of animals are processed in slaughterhouses under mandatory oversight by meat inspectors, though infrastructure and enforcement vary^40,50^.

Our study focused on three major slaughterhouses sourcing from and serving the local market: Kimana (−2.6458, 37.4710), Loitokitok (−2.8775, 37.5270), and Rombo (−2.7489, 37.8583). When the El Niño rains from November of 2023 destroyed the road to Rombo, animals were instead sampled at the nearby Illasit slaughterhouse (−2.7714, 37.8650). All of these sites are class C slaughterhouses under the Kenya Meat Control Act Cap. 356, slaughtering < 6 bovines per day, though some butchers engage in wider distribution^51^.

### Sample size calculation

Five consecutive cross-sectional surveys were carried out across 13 months. Sample size for each survey was calculated to estimate RVFV seroprevalence, assumed to be between 13–15%^16,52^, using the formula where Z = 1.96 (95% CI), p = expected proportion, and d = 0.05. Each animal was considered an independent presentation, so no design effect correction was applied.

$$n=\frac{{\mathrm{Z}}^{2}\cdot \mathrm{p}\cdot \left(1-\mathrm{p}\right)}{{d}^{2}}$$

Requiring between 174–196 animals in each sampling period, we sampled five times, for 15–20 days each following the schedule outlined in Table 3.

**Table 3:** Sampling dates for each period and the total number of samples.

Sampling period	Dates of sampling	Total number of samples
A	May 2^nd^26^th^ 2023	206
B	July 31^st^ Aug 16^th^ 2023	213
C	Nov 20^th^ – Dec 14^th^ 2023	178
D	Jan 17^th^ – Feb 15^th^ 2024	172
E	May 14^th^ – June 21^st^ 2024	186
All (A-E)	May 2^nd^ 2023 – June 21^st^ 2024	955

### Sampling and data collection

Cattle, sheep, and goats were sampled at slaughter during exsanguination with blood collected in a 15 mL conical tube^33^ by meat inspectors and assistants who recorded the metadata including species, estimated age via dentition, animal origin, if they were purchased from a market, how many animals they may have arrived with, and the means of transport. Routine post-mortem inspection findings were linked to each sample. At the smaller slaughterhouses (Loitokitok, Rombo, Ilasit), all animals slaughtered on a given day were sampled; at Kimana every second animal was sampled to ensure time for accurate data linkage.

Laminated data cards (Supplementary S2) were filled daily and digitized in the laboratory space using ONA software. Samples were transported to the Loitokitok One Health laboratory in Loitoktok town using the meat inspector’s usual means of public transport. Working directly with meat inspectors allowed us to link samples to post-mortem lesions, as Kenyan law requires inspection of all carcasses, and organs are meticulously matched.

### Laboratory analysis

#### Sample storage

Samples from the slaughterhouse were held at 4 °C overnight to ensure clotting before centrifugation to separate serum that was aliquoted. Over the sampling period, serum was stored at −40 °C in the Loitokitok One Health laboratory in Loitoktiok, Kenya. When the sampling period was completed, samples were transferred to Nairobi and thence to the −80 °C freezer at ILRI in Nairobi, Kenya where they were held until antibody testing was carried out.

### Antibody detection

All serum samples were tested for IgG and IgM antibodies using commercially available test kits (ID Vet, Grables, France). Protocols were followed verbatim, and all IgM-positive samples were repeated to confirm results.

### RNA Extraction and RT–qPCR detection of RVFV

RNA was extracted from IgM-positive serum samples using the TANBead OptiPure kit (TANBead Technology, Taiwan) per the manufacturer’s directions. RVFV RNA was then detected with a one-step RT–qPCR targeting the L-segment using published primers^53^ and probe, run on a QuantStudio 5 system under standard cycling conditions^54,55^.

### Data analysis

Metadata were matched to unique sample IDs, unmatched samples (n = 60) were excluded from analysis. The two primary outcomes were individual animals’ exposure status (IgG) and evidence of recent infection (IgM), as binary outcomes. We used Chi-square or Fisher’s exact tests to assess categorical statistical associations, and the Cochran-Armitage test was used for ordinal variables. Seroprevalence and age distribution were visualized across sampling periods. While the primary aim of this study was surveillance and descriptive statistics of the samples collected, we also performed an exploratory multivariable logistic regression to evaluate the variables that remain independently associated with IgG seropositivity after adjustment. All analyses described below were conducted in R (version 4.4.3) using RStudio, and all visualizations were generated with ggplot2**.**

### Force of infection (FOI) estimation

To estimate changes in transmission intensity over time, we calculated the force of infection (FOI) by combining adjacent sampling periods to increase sample size for age-stratum and ensure seroprevalence increases with age as required by this modelling approach. We applied a standard catalytic model that assumes a constant FOI for the entire sampling period^22,56^. Seroprevalence at age a, P(a), was modelled as:

$$P\left(a\right)= 1 - {e}^{\left\{- \lambda a\right\}}$$

where λ is the FOI. The model was linearized using the log-transformed complement of seroprevalence, and fit using a weighted linear regression in the form:

$$ln\left(1 - P\left(a\right)\right)= -\lambda a$$

The FOI was estimated as the negative of the regression slope. Age groups with very small sample size or an overinflated (> 90%) seroprevalence were excluded and the FOI estimates were compared with and without the youngest age group. These comparisons ensured FOI estimates that were not driven by outliers.

### Spatial clustering of RVFV exposure and associations with post-mortem lesions

#### Post-mortem lesions and spatial data preparation

Lesion data were aggregated by slaughterhouse and organ, and associations with IgG status were tested using Chi-square or Fisher’s exact tests.

The animal origin data collected at slaughter was reported by the stakeholders during data collection and used to determine if there were spatial significance in our outcome data. All reported origins were georeferenced and assigned GPS coordinates by K.G. and A.R. Reported origins were georeferenced using centroids or nearby landmarks and closely spaced points within 1,000 m were combined. Only animals with a reported and georeferenced origin were included in spatial analysis. All spatial layers were transformed to a common geographic coordinate system (WGS 84, EPSG:4326).

#### Spatial cluster visualization and analysis

To explore variability in spatial patterns of RVFV IgG seropositivity, we first visualized the origin distribution. Animal origin location data were aggregated to calculate the total number of animals sampled, the number seropositive, and the proportion seropositive at each location, and these were overlaid on the Loitokitok sub-county administrative boundary (GADM level 3 for Kenya, https://gadm.org/data.html).

Spatial analyses considered whether RVFV exposure or major post-mortem lesions were clustered in specific hotspots in the study site. Coordinates were transformed to UTM Zone 37S for distance-based calculations. Global spatial autocorrelation of IgG seropositive cases and the most common lesion types were assessed using join count tests for binary outcomes, with neighbours set at 5 km using the spdep package in R.

To further assess location variation in seropositivity, logistic regression was used to test associations between IgG status and locations overall and for each sampling period. Only locations that provided at least five samples were included in these analyses.

## Ethics approval

This study was approved under the International Livestock Research Institute (ILRI) Institutional Animal Care and Use Committee (IACUC) (IACUC2022-47/1) and ILRI Institutional Research Ethics Committee (ILRI-IREC2022-69) for our engagement with animal owners to request origin data. All research procedures were carried out in accordance with ILRI IACUC guidelines and included in the NACOSTI licence to K.G. (NACOSTI/P/24/34,088).

## Supplementary Information

Supplementary Information 1.

Supplementary Information 2.

## Data Availability

The dataset used to generate this analysis is available at 10.5281/zenodo.17078167. Other associated data, including the georeferenced locations, is available on reasonable request by writing to the corresponding author.
