# Supplementary material for: Unreported Rift Valley fever virus circulation during 2023–2024 El Niño event detected by slaughterhouse-based surveillance in southern Kenya
Source: Sci Rep. 2026 Mar 19;16:14123. doi: 10.1038/s41598-026-44706-y (PMC13136298; doi:10.1038/s41598-026-44706-y)
Supplement: Supplementary file 1 — Supplementary Information 1. [file 41598_2026_44706_MOESM1_ESM.pdf]

Owner's name_________________Sample ID_________________ SH: ____________Date______________________

1. Species
   - ☐ Cattle
   - ☐ Sheep
   - ☐ Goat
2. **Number** received with herd_______
3. **Origin** of this animal________________________________
4. Vehicle transport?
   - ☐ No
   - ☐ Yes ____________________________
5. Was it purchased at a **market**?
   - ☐ No
   - ☐ Yes ____________________________
6. How many adult **teeth** does this animal have?
   - ☐ 0 (very young_
   - ☐ 2
   - ☐ 4
   - ☐ 6
   - ☐ 8
   - ☐ 0 (Old animal teeth fallen out)

Lesions?

Overall appearance

❑ Abnormal____

❑ Condemned (Full or Partial) KES___________

❑ Normal

- Heart lesion?
  - ❑ Yes____
  - ☐ Condemned (Full or Partial) KES______________
- Spleen
  - ❑ Yes____
  - ❑ Condemned (Full or Partial) KES______________
- Muscle/Head
  - ❑ Yes____
  - ☐ Condemned (Full or Partial) KES______________
- **Liver lesion**
  - ☐ Yes____
  - ❑ Condemned (Full or Partial) KES______________
  - ❑ Target liver lesion?
    - Distribution__________________________________
    - Size____
    - Color/texture_________________________________
- Kidney
  - ☐ Yes____
  - ❑ Condemned (Full or Partial) KES______________
  - ❑ Target kidney lesion?
    - Distribution__________________________________
    - Size____
    - Color/texture_________________________________
- Lungs
  - ☐ Yes____
  - ❑ Condemned (Full or Partial) KES______________
  - ❑ Target lung lesion?
    - Distribution__________________________________
    - Size____
    - Color/texture_________________________________
