# Supplementary material for: Unreported Rift Valley fever virus circulation during 2023–2024 El Niño event detected by slaughterhouse-based surveillance in southern Kenya
Source: Sci Rep. 2026 Mar 19;16:14123. doi: 10.1038/s41598-026-44706-y (PMC13136298; doi:10.1038/s41598-026-44706-y)
Supplement: Supplementary file 2 — Supplementary Information 2. [file 41598_2026_44706_MOESM2_ESM.docx]

**Supplementary Table S1: Lesion characteristics by organ system**

Total lesions n=143 as a binary Yes/No

| **Organ**  **system** | **Lesion Type** | **N=** | **% in organ system** | **Notes** |
| --- | --- | --- | --- | --- |
| **Liver** | Liver overall | 86 |  |  |
|  | Fluke damage/tracks | 53 | 61.6 |  |
|  | Hepatitis | 10 | 11.6 |  |
|  | Stellesia hepatica | 5 | 5.8 |  |
|  | Abscess | 4 | 4.7 |  |
|  | Calcified cysts | 3 | 3.5 |  |
|  | Melanosis | 3 | 3.5 |  |
|  | Milky spots | 3 | 3.5 |  |
| **Spleen** | Spleen overall | 13 |  |  |
|  | Splenomegaly | 10 | 76.9 |  |
|  | Abscess | 3 | 23.1 |  |
| **Lung** | Lung overall | 66 |  |  |
|  | Emphysema | 30 | 45.5 |  |
|  | Cysts (total) | 13 | 19.7 | 9 specifically hydatid cysts |
|  | Haemorrhage | 8 | 12.1 |  |
|  | Blood inhalation | 6 | 9.1 |  |
|  | Pneumonia | 5 | 7.6 |  |
| **Kidney** | Kidney overall |  |  |  |
|  | Hydronephrosis | 9 | 30.0 | 8 from Loitokitok |
|  | Nephritis | 9 | 30.0 |  |
|  | Congenital cysts | 8 | 26.7 |  |
|  | Infarcts | 3 | 10.0 |  |
| **Heart** | Heart overall | 4 |  |  |
|  | Haemorrhage | 2 | 50.0 |  |
|  | Pericarditis | 1 | 25.0 | Total 4 cases |
|  |  | 1 | 25.0 |  |

Notes:

This study had 3/143 total carcass abnormalities including two emaciated animals and one with diffuse pustules and jaundice.

All hydronephrosis cases were identified at the Loitoktiok slaughterhouse except for one.
